# Microbial tolerance engineering for boosting lactic acid production from lignocellulose

**DOI:** 10.1186/s13068-023-02334-y

**Published:** 2023-05-11

**Authors:** Wenwen Shan, Yongli Yan, Yongda Li, Wei Hu, Jihong Chen

**Affiliations:** 1 Department of Biophysics, Institute of Modern Physics, Chinese Academy of Sciences, 509 Nanchang Road, Lanzhou, 730000 People’s Republic of China; 2 University of Chinese Academy of Sciences, Beijing, People’s Republic of China; 3 College of Food Science and Engineering, Gansu Agricultural University, Lanzhou, People’s Republic of China

**Keywords:** Pretreatment, Inhibitor, Lactic acid, Tolerance modification

## Abstract

Lignocellulosic biomass is an attractive non-food feedstock for lactic acid production via microbial conversion due to its abundance and low-price, which can alleviate the conflict with food supplies. However, a variety of inhibitors derived from the biomass pretreatment processes repress microbial growth, decrease feedstock conversion efficiency and increase lactic acid production costs. Microbial tolerance engineering strategies accelerate the conversion of carbohydrates by improving microbial tolerance to toxic inhibitors using pretreated lignocellulose hydrolysate as a feedstock. This review presents the recent significant progress in microbial tolerance engineering to develop robust microbial cell factories with inhibitor tolerance and their application for cellulosic lactic acid production. Moreover, microbial tolerance engineering crosslinking other efficient breeding tools and novel approaches are also deeply discussed, aiming to providing a practical guide for economically viable production of cellulosic lactic acid.

## Background

Polylactic acid (PLA), a kind of biodegradable bioplastics, has the great potential to partially replace petroleum-derived plastics [1, 2], and also increases the demand for its monomers such as optically pure l- and d-lactic acids [3]. About 50% lactic acid (LA) in global market is expected to produce PLA by 2025 [2]. To date, the production of LA is usually based on the microbial fermentation using carbohydrates from food sources [4, 5], but accelerating competition with food supplies [6]. Thus, the application of renewable lignocellulosic biomass (such as agricultural and forest residues, energy crops, and cellulosic wastes) for LA production through fermentation would contribute to be a promising scheme to alleviating food supply crisis [7–10].

Currently, using lignocellulosic biomass as a non-food feedstock platform for the production of LA, lignocellulosic biomass needs to be pretreated by different pretreatment methods [11], which can destroy lignocellulose recalcitrance and remove lignin and hemicellulose. Different pretreatment methods have different technological characteristics and challenges for downstream LA fermentation process (Table 1). However, there is no doubt that the inhibitors (such as furan derivatives, weak acids, and phenolic compounds) derived from the degradation of lignocellulose biomass might be generated after the pretreatment process by the majority of pretreatment methods [6, 12, 13]. One of the main difficulties for LA fermentation production from lignocellulosic biomass could be the toxic effect of a variety of inhibitors to LA production strains. These inhibitors can adversely affect microbial cell viability, decrease feedstock conversion efficiency and increase production costs [14, 15]. Of all the practical approaches to overcome these inhibitors effect, the most competitive approach is to improve the tolerance robustness of LA strains.

**Table 1:** Mechanism of action, advantages and disadvantages of different pretreatment methods

Approach	Mode of action	Advantages	Disadvantages	References
Mechanical extrusion	Reducing the particle size and space structure of biomass	∙ No inhibitor formation ∙ Environmental friendliness ∙ Easy control	∙ High energy	[16, 17]
Milling/grinding	Increasing specific surface area and reducing the crystallinity of cellulose	∙ No inhibitor formation like HMF and levulinic acid ∙ High effectiveness for enzymatic hydrolysis	∙ High energy ∙ Effect is limited when no chemical agents are used	[17–19]
Microwave pretreatment	The expansion of biomass via rapid and volumetric heating	∙ Short reaction time ∙ Easy operation ∙ Minimum generation of byproducts	∙ High cost ∙ Effect is limited when no other pretreatment are used	[16, 20]
Ultrasound	Cleaving the α-O-4 and β-O-4 linkages in lignin	∙ Reducing pretreatment time and enzyme consumption	∙ Effect is limited when no other pretreatment are used	[16, 21]
Acid	Reducing the crystallinity of cellulose, releasing oligomers and carbohydrates	∙ Simple method ∙ No thermal energy demand	∙ Produce inhibitors ∙ Corrosive properties ∙ Environmental concerns	[22, 23]
Alkali	Removing lignin and part of the hemicellulose, and reducing cellulose crystallinity	∙ Efficiency in obtaining cellulose pulp ∙ Low energy consumption	∙ Formation of irrecoverable salts ∙ Toxic compounds generation	[22, 24]
Ionic liquids	Reducing cellulose crystallinity and partial removing hemicellulose and lignin	∙ Less energy ∙ Easy to operate	∙ High cost of recovery and recycling ∙ Toxic compounds generation	[16, 17, 25]
Organic solvent	Solubilizing hemicellulose and extracting lignin	∙ High penetration efficiency ∙ Recycling and reuse	∙ Expensive investments, ∙ Environmentally unfriendly ∙ High inhibitory products	[16, 26]
Deep eutectic solvents	Removing lignin and hemicelluloses	∙ Green solvent ∙ Highly biodegradable	∙ High pretreatment temperatures ∙ Instability	[16, 20, 25]
Oxidative pretreatment	Reducing the crystallinity of cellulose	∙ Environmentally friendly ∙ Low toxic compounds generation ∙ Mild conditions	∙ High cost	[20, 23, 27]
Biological pretreatment	Decomposing lignin and hemicellulose	∙ Mild conditions ∙ Low power consumption	∙ Low efficiency	[21, 28]
Steam explosion	Lignin softening and particle size reduction	∙ Low requirement of hazardous chemicals ∙ High sugar recovery	∙ Produce inhibitors ∙ High energy ∙ High pressure	[16, 29, 30]
Ammonia Fiber Expansion	Reducing the crystallinity of cellulose and removing lignin	∙ High efficiency and selectivity for reaction with lignin	∙ Low efficiency for softwood ∙ Cost of ammonia	[16, 20, 31]

Fortunately, some innovative microbial engineering methods can enrich the desired tolerance robustness microbe to overcome the toxic effect of the inhibitors including random mutagenesis and screening, adaptive laboratory evolution, metabolic engineering, etc. These microbial engineering strategies also cross-link with each other to further improve the construction efficiency of tolerance robustness microbe. Within this review, we summarize the current significant progress using these microbial engineering strategies to construct LA strains with strong tolerance against the inhibitors derived from the processes of biomass pretreatment. More specially, future perspectives on improving the biomass utilization economics for cellulosic LA production are also presented.

### Inhibitory compounds derived from the treatment of lignocellulosic biomass and their molecular toxic mechanisms

Destroying lignocellulose recalcitrance of lignocellulosic biomass via different pretreatment methods is a critical step for downstream efficient enzymatic saccharification [32] and high LA production [33]. However, some toxic inhibitory compounds are usually generated after the pretreatment [15, 34–36], mainly including three major groups such as phenolic compounds generated from the breakdown of lignin components, furan derivatives (e.g., furfural and 5-hydroxymethyl furfural [HMF]) generated from the dehydration of pentose and hexose sugars and short-chain aliphatic acids (e.g., acetic acid generated from the deacetylation of hemicellulose and lignin, formic acid generated from the degradation of furans, and levulinic acid generated from the degradation of HMF). In addition, some pretreatment solvents and inorganic salts are another source of toxic inhibitory compounds in lignocellulosic hydrolysates from pretreatment process or the corrosion of pretreatment equipment [36, 37].

Different inhibitory compounds have distinct molecular functional groups and molecular toxic mechanisms on host microbial strains [38]. For instance, furan derivatives usually caused multiple toxicities to microbial strains [35, 39, 40] including the inhibition of glycolytic and fermentative enzymes, disrupting cellular energy and decreasing intracellular ATP and NAD(P)H levels, increasing free radical generation and the damage of cell membrane, etc. Weak acids also showed microbial toxicity [12, 37, 38, 41], which can disrupt the proton gradient of the membrane and uncouple of the proton pump, destroy membrane integrity and intracellular redox homeostasis, induce anion accumulation, etc. Phenolic compounds mainly destroyed cellular membrane for the hydrophobicity, increase membrane fluidity, promoting ROS accumulation [37, 42], etc. Thus, these toxic inhibitory compounds significantly affected the downstream cellulosic microbial fermentation efficiency by suppressing cell growth or catalytic action of cellulolytic enzymes.

### Evaluation of the effect of hydrolysate inhibitors on LA-producing strains

In order to help elucidate inhibitor tolerance mechanisms and develop robust LA strains, evaluation of the effect of hydrolysate inhibitors on LA production is critical. For instance, the effects of inhibitory hydrolysate compounds (such as 2-furfural, vanillin, formic acid and acetic acid) on *Bacillus* sp. P38 LA fermentation have been investigated [5]. The results demonstrated *Bacillus* sp. P38 showed strong tolerance capacity to 2-furfural (up to 10 g/L) and excellent LA fermentation performance (below 6 g/L 2-furfural). It was also observed that *Bacillus* sp. P38 was capable of degrading 2-furfural. Based on transcriptome analysis results, differentially expressed alcohol dehydrogenase genes and short-chain dehydrogenase/reductase genes may be the key to strong 2-furfural tolerance of *Bacillus* sp. P38 [43]. Other researches has also proved that overexpression of some short-chain dehydrogenase/reductases genes could enhance the strain tolerance of furfural [44], possibly because short-chain dehydrogenase/reductases genes could degrade furfural into the less toxic furfuryl alcohol. Similarly, Qiu et al. also reported a robust adapted *Pediococcus acidilactici* XH11 with 100% improvement of D-LA production using undetoxified acid-pretreated corncob slurry [3]. The adapted strain enabled the toxic four typical aldehyde inhibitors (furfural, HMF, vanillin, and 4-hydroxybenzaldehyde) to be converted more efficiently compared to the parental strain, leading to lower cytotoxicity and higher D-LA titers. These studies suggested that the enhanced conversion of toxic inhibitor into less toxic intermediates with engineered microbial cell factories could reduce fermentation cost by improving LA titers and/or by reducing fermentation time.

### Construction of tolerant LA strains based on innovative microbial engineering methods

To alleviate the toxic effect of hydrolysate compounds, many efforts including screening of new tolerant strains [5, 45], detoxification processes [46, 47], advanced process engineering strategy [48], and seed precultivation [49, 50], have been developed. For instance, in fed-batch fermentation, a newly isolated *Bacillus* sp. P38 with high 2-furfural tolerance, produced 180 g/L LA with the productivity of 2.4 g/L/h from corn stover hydrolysate treated by a traditional acid [5]. In another study, a newly isolated *Bacillus coagulans* strain IPE22 also showed good tolerance to some inhibitors (such as furans, acetate, and sulfuric acid) from wheat straw hydrolysate treated by dilute sulfuric acid, resulting in 46.12 g LA production from 100 g dry wheat straw via simultaneous saccharification and co-fermentation (SSCF) [51]. After the detoxification by *Amorphotheca resinae* ZN1, the fermentable sugars (both poly- and mono-saccharides) were well retained and residual toxic phenolic aldehydes (4-hydroxybenzaldehyde, 0.1 ± 0.0 mg/g dry feedstock matter (DM); vanillin, 0.2 ± 0.0 mg/g DM; syringaldehyde, 0.5 ± 0.0 mg/g DM) were at minor level in the pretreated lignocellulose. Thus, a high L-LA titer (129.4 g/L) and minor residual total sugars (~ 2.2 g/L) were obtained from pretreated wheat straw [52]. Based on seed precultivation strategy, the inhibitory effects of acid-catalysed sream explosion wheat straw hydrolysate (mainly containing 3.8 g/L acetic acid, 4.0 g/L furfural, 1.4 g/L HMF, etc.) were reduced and the LA productivity was increased [50]. However, because of the sugar loss, complex processes, residual toxic phenolic aldehydes after detoxification or low LA production rate, these efficient methods were not always cost-efficient for cellulosic LA production. Thus, construction of tolerant LA strains will further improve economic feasibility of cellulosic LA production.

**Fig. 1:**
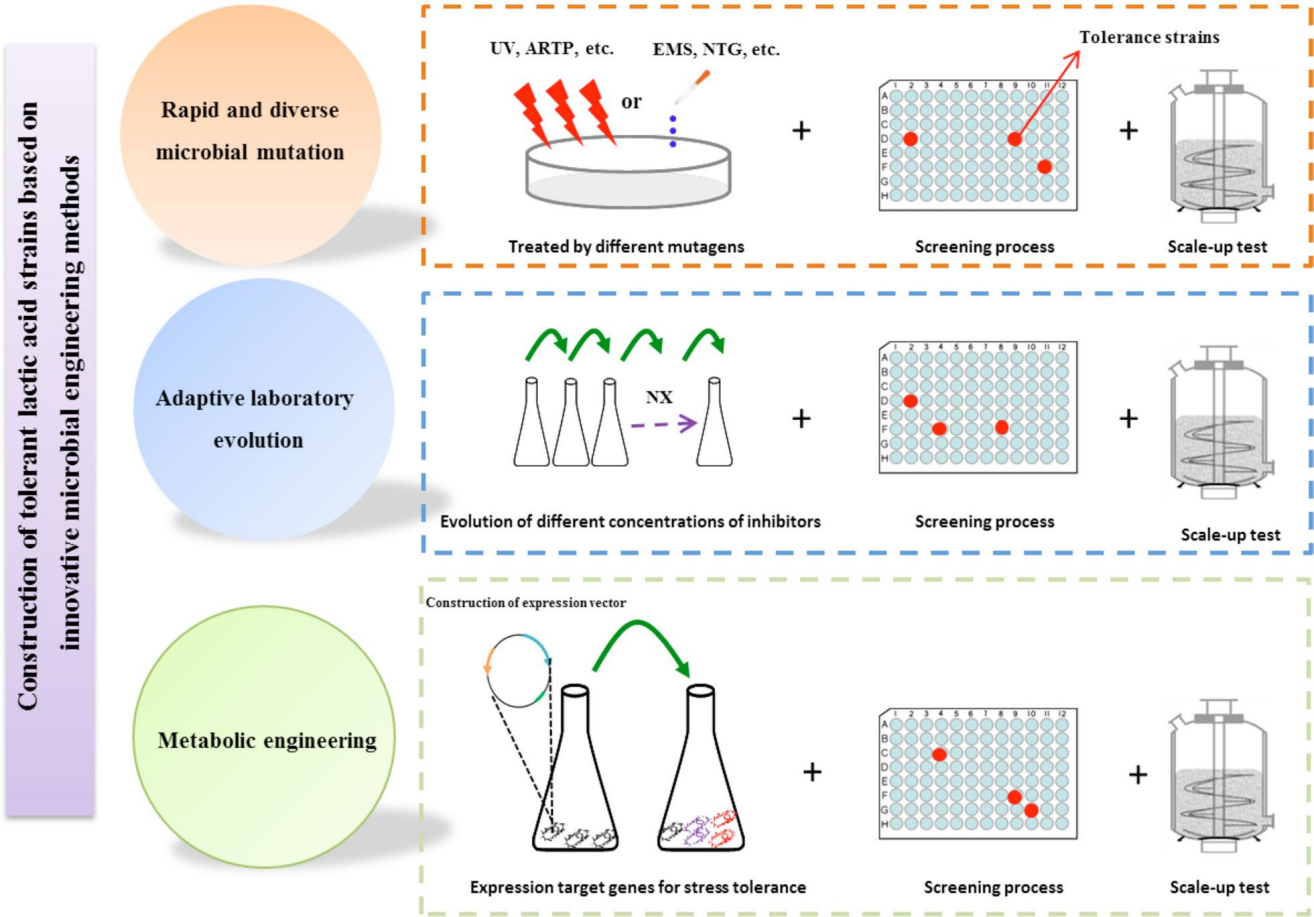
Construction of tolerant LA strains based on innovative microbial engineering methods

Several valuable microbial engineering methods for the construction of tolerant microbe has been developed [37, 53, 54], such as random mutagenesis, adaptive laboratory evolution, genome shuffling, global transcription machinery engineering and metabolic engineering. Although genome shuffling and global transcription machinery engineering were the powerful tools to construct stress tolerance microbe to inhibitors derived from the degradation of lignocellulose biomass [53, 55, 56], these two useful strategies have been rarely applied in the modification of highly tolerant cellulosic LA strains. Thus, we mainly focused on the other three microbial engineering methods to improve the tolerance ability of LA strains against inhibitors as following (Fig. 1):

### Strain tolerance modification based on random mutagenesis

Random mutagenesis including physical and chemical mutagens combined with appropriate screening strategies provides a classic approach for strain tolerance improvement (Table 2). Among these random mutagenesis methods, heavy ion mutagenesis and atmospheric and room temperature plasma (ARTP) show powerful industrial application for microbial strain improvement with desired phenotype [57, 58]. These new mutagenesis approaches can accelerate the acquisition of highly tolerance strains. For example, in the study of Wu et al. two obtained *Zymomonas mobilis* mutants both showed enhanced acetic acid tolerance after a multiplex ARTP mutagenesis [12]. Mutagenesis strategy is also used to improve tolerance robustness of microbial cell factories to inhibitors for cellulosic LA production. For instance, Jiang et al. employed ARTP and evolution strategy to treated *B. coagulans* NL01 and obtained a tolerance mutant strain *B. coagulans* GKN316, which exhibited an significantly increase of LA accumulation by 1.9 times to 45.39 g/L from undetoxified acid-catalyzed steam-exploded corn stover hydrolysate compared to the results from parental strain NL01 [59]. It was also observed that *B. coagulans* GKN316 could effectively degrade toxic inhibitors (furan derivatives and phenolic compounds) to the less toxic corresponding alcohols, primarily leading to higher LA accumulation.

**Table 2:** Strain tolerance modification based on random mutagenesis

	Against inhibitors	Products	Microorganism	References
UV mutagenesis following adaptation	Acetic acid, furfural and vanillin	Ethanol	*Scheffersomyces stipitis*	[60]
UV mutagenesis	Furfural	Ethanol	*Spathaspora passalidarum*	[61]
UV mutagenesis	Acetic acid and furfural	Ethanol	*Scheffersomyces shehatae*	[62]
UV mutagenesis	Acetic acid and HMF	Ethanol	*Pichia stipitis*	[63]
Low-energy ion implantation	*p*-coumaric acid, ferulic acid, 4-hydroxybutyl acrylate, vanillic acid, syringaldehyde	Acetone/butanol/ethanol	*Clostridium beijerinckii*	[64]
ARTP following adaptation	Furfural, HMF, vanillin, syringaldehyde and p-hydroxybenzaldehyde	LA	*B. coagulans*	[59]
ARTP	Formic acid, acetic acid, levulinic acid, HMF, vanillin	Lipid	*Rhodosporidium toruloides*	[65]
Multiplex ARTP	Acetic acid	Ethanol	*Z. mobilis*	[12]

### Strain tolerance modification based on adaptive laboratory evolution

As a microbial engineering method, adaptive laboratory evolution is a promising tool to improve microbial tolerance to environmental stresses [66]. Many successful cases have been reported via this strategy such as increasing chemicals stress, acids stress and osmotic pressure [66–68]. This strategy has also been widely used to enhance tolerance robustness of microbial cell factories to inhibitors for cellulosic LA production. For instance, mutant *P. acidilactici* XH11 was obtained after 111 days’ long-term adaptive evolution, showing enhanced inhibitors tolerance and D-LA production (61.9 g/L) using undetoxified whole slurry of pretreated corncob compared to the parental strain, primarily due to its improved degradation capacities of four typical aldehyde inhibitors [3]. In another study, a mix-milling of biomass and P_2_O_5_ pretreatment method was developed, showing less inhibitory compounds generation compared to conventional methods. Then, a domesticated *Pediococcus pentosaceus* strain B was obtained, showing superior inhibitors tolerance (17.1 g/L acetic acid, 12.5 g/L 5-HMF, 11.9 g/L guaiacol and 11.5 g/L furfural) and the corresponding self-detoxification ability. Finally, based on combination of P_2_O_5_ pretreatment and strain domestication, LA concentration of 29.8 g/L, 31.1 g/L, and 46.2 g/L were produced by fed-batch fermentation using undetoxified corn stalk, corn stalk residue and rice husk residue, respectively [69].

### Strain tolerance modification based on genetic and metabolic engineering strategy

Based on the metabolic engineering technology, the tolerance of microbial cell factory is largely boosted. As a result, the engineered microbial cell factory has better cell viability and metabolite yield when exposed to the inhibitors derived from the degradation of lignocellulose. In general, genetic and metabolic engineering strategy is used for constructing tolerant microbial cell factories, mainly based on in situ detoxification, efflux pumps, stress responses and membrane engineering [37]. Among them, converting toxic inhibitors into less toxic intermediates is a common strategy [54]. For example, NADH-dependent oxidoreductase (FucO) belonging to short-chain dehydrogenase/reductases can degrade inhibitor 2-furfural into the less toxic furfuryl alcohol and overexpressed FucO gene in *E. coli* did lead to increased furfural tolerance [43, 44].

According to this strategy, some improved microbial cell factories have been constructed to enhance the strain's ability to tolerate inhibitors and also increase the production of cellulosic LA. For instance, Qiu et al. overexpressed a short-chain dehydrogenase *CGS9114_RS09725* from *Corynebacterium glutamicum* in *P. acidilactici*, showing enhanced vanillin degradation rate. Finally, based on SSCF process, engineered *P. acidilactici* can produce 115 g/L D-LA production with the productivity of 1.6 g/L/h and overall yield of 61.1% using dry acid pretreated and biodetoxified corn stover [70]. With the same strategy, Qiu et al. also overexpressed another oxidoreductase gene *ZMO1116* from *Z. mobilis* in *P. acidilactici* via degradation of *p*-benzoquinone into less toxic hydroquinone (HQ) [71], achieving a rapid accumulation of D-LA (123.8 g/L) using dry acid pretreated and biodetoxified corn stover as a feedstock.

### Future perspectives

As a matter of fact, pretreatment and fermentation are necessary processes for LA production. However, toxic inhibitors are usually generated by the majority of pretreatment methods [6], which is one of the main drawbacks for LA fermentation. To circumvent the drawback, several potential approaches has been reported including modifying cell walls of crops or energy crops, developing efficient and cheap detoxification means, developing process-oriented approaches, and constructing tolerance robustness of microbial cell factories [6, 33, 72]. Currently, detoxification process is a key process to produce high concentration of biochemicals using cellulosic hydrolysate and a short biological detoxification process is the most promising [70], which can minimize xylose consumption and remove most of the inhibitors including furfural, HMF and acetic acid. However, a certain concentration of the toxic phenolic aldehydes remain in the cellulose hydrolysate after a short biological detoxification process [73, 74], which still negatively affect the fermentation performance of LA strains. Thus, construction of high-tolerance LA strains is still beneficial for downstream LA production cost reduction.

Although there are many microbial breeding methods as mentioned above that are applied to the construction of tolerant microbial cell factories for cellulosic LA production, poor construction efficiency are still limited. Thus, improving the construction efficiency will be necessary. In addition, novel approaches could further improve the economic competitiveness of cellulosic LA production. We believe that these limited construction efficiency and economic competitiveness can be further addressed by employing suitable technical strategies as following.

#### Improving the construction efficiency of microbial cell factories with tolerance robustness for cellulosic LA production

Mutagenesis screening strategy with traditional chemical and physical mutagens is one of the classic approaches for constructing high-tolerance microbes, and is also used to constructing high-tolerance LA-producing strains, while the limited drawback is low screening efficiency because a large number of candidate mutants are tested based on shake flask and this process is a time consuming. Thus, high-throughput screening strategy will be proposed to overcome this drawback and improve the construction efficiency of LA-producing strain with high tolerance to inhibitors in future. According to high-throughput screening methods [75, 76] including multilabel plate reader screening strategy, fluorescence-activated cell sorting screening strategy and microfluidics screening strategy, the screening process shows high screening efficiency, more efficient automated operation, fewer manual participation, and lower sample volumes [76]. For instance, based on a deep-well microtiter plate, a new high-throughput screening strategy was built, and high L-LA *B. coagulans* mutant IIIB5 was obtained after ARTP mutagenesis [77], showing faster LA productivities (46.10%) than that of parental strain. In another study of Zhu et al. [78], an ultrahigh fluorescence-activated cell sorting system based on a pH fluorescence biosensor was used to screen high LA *B. coagulans* mutants after ARTP mutagenesis. Finally, a mutant E11 was also obtained, which exhibited an increase of LA by 52% to 76 g/L compared to the results noted by parental strain.

In terms of adaptive laboratory evolution, repetitive manual transfer and difficult parallelization are the main drawbacks [66] for constructing high-tolerance LA-producing strains. Recently, several multiplexed automated culture systems (such as iBioFAB, milliliter-scale Mini Pilot Plant, Omnistat and eVOLVER) [79–82] have been developed for ALE application, and the fermentation parameters including OD, temperature, pH and dissolved oxygen, can be monitored, leading to significantly improved the automation and parallelization. Especially, an integrated platform named microbial microdroplet culture (MMC), exhibited automated and high-throughput properties for microbial cultivation and ALE [66, 83]. In this process, up to 200 replicate droplets of 2.00 µL volume can be cultured simultaneously for tolerance domestication. For example, a high D-sorbitol and temperature tolerance was a critical bottleneck for the conversion of D-sorbitol into L-sorbose in *Gluconobacter oxydans*. Thus, a high-tolerance evolved mutant MMC10 to 300 g/L of D-sorbitol and 40 ℃ temperature, was screened based on the MMC strategy [84], showing significantly increased tolerance improvements compared to the results of parental strain. There is no doubt that these advanced ALE tools will boost the construction efficiency of LA-producing strain with high tolerance to inhibitors in future.

The lack of efficient tolerance-related genes for metabolic engineering is the main drawback for constructing tolerant microbial cell factories for cellulosic LA production. Based on omics tools (transcriptomics, proteomics and metabolomics), systems biology strategy provide a new window to address this drawback [85]. On one hand, tolerance-related genes can be identified via omics analysis from strains exposed to different inhibitors stresses cultivation (such as 2-furfural, phenolic aldehydes and acetic acid). For instance, several different gene expressions involved in alcohol dehydrogenase and short-chain dehydrogenase/reductase were screened for responding to 2-furfural tolerance in *B. coagulans* P38 via transcriptome analysis [43]. With the same strategy, three encoded reductases genes (such as ZMO1696, ZMO1116, and ZMO1885) were identified after exposing *Z. mobilis* ZM4 to phenolic aldehyde inhibitors, and overexpressed these three genes in *Z. mobilis* ZM4 significantly boosted its phenolic aldehydes tolerance and ethanol production [86]. On the other hand, the tolerance-related genes identification is also performed based on the mutants with tolerance improvement. In this case, different omics tools are used to identify different expression genes levels in improved tolerance mutants exposed to different inhibitors stresses, and tolerance-related genes are then acquired. For instance, in one study, based on the transcriptome strategy in evolved *B. coagulans* CC17A mutant, highly up-regulated oxidoreductases and phenolic acid decarboxylase genes were identified for inhibitors-tolerance modification and LA accumulation [87]. It is worth noting that highly precise and efficient CRISPR/Cas9 gene editing tool for metabolic engineering strategy has been developed to modify L-LA optical purity of LA strain [88]. Thus, metabolic engineering based on these newly discovered tolerance-related genes and efficient CRISPR/Cas9 gene editing tool could be implemented to further accelerate the construction of high inhibitors-tolerance LA strains.

#### Modified cell walls of crops or energy crops for cellulosic LA production

Key obstacle of lignocellulosic biomass utilization for cellulosic biochemicals production including LA via microbial conversion is the poor enzymatic saccharification [89]. The pretreatment process can enhance enzymatic digestibility of lignocellulosic biomass effectively [11], but the high pretreatment cost and inhibitors derived from the processes of biomass pretreatment seriously reduce the downstream LA production economy. Thus, on one hand, developing new pretreatment strategies or modifying current pretreatment strategies and strain improvement technologies are efficient methods to boosted cellulosic LA production via alleviating these inhibition effects. On the other hand, breeding with modified cell walls of crops or energy crops varieties to reduce lignocellulosic recalcitrance and improve enzymatic saccharification efficiency, is also absolutely necessary. These modified crops or energy crops can be easier pretreated by some mild pretreatment methods [72] or a direct enzymatic hydrolysis [32], resulting in lesser or no inhibitors generation. For instance, construction of *OsGH9B1* and *OsGH9B3* transgenic rice lines with modified cell wall compositions [90], showed improved enzymatic hydrolysis, leading to high bioethanol production. Similar strategy was also tried by Wu et al. [91]. In another study, *miscanthus* mutant was also constructed via heavy ion mutagenesis, showing lower lignin content, higher cellulose content and higher saccharification efficiency compared with the parental plant [92]. In breeding process, innovations such as novel mutagenesis technology, marker-assisted selection technology and genome-editing technology will speed up breeding of modified cell walls of crops or energy crops varieties [92–94].

#### Synergistic microbial consortia for cellulosic LA production

The lignocellulosic hydrolysate treated by the majority of pretreatment methods generally contains different types and concentrations of inhibitors and a mixture of pentose sugars (C5) and glucose (C6), while the mixture of C5 and C6 sugars and these inhibitors in hydrolysate both result in low cellulosic LA productivity due to the carbon catabolite repression effect (CCR) and toxic effects of inhibitors [95]. To overcome these challenges, microbial consortia provide a new way to solve these issues. For instance, a thermophilic microbial consortium DUT50 (50 ℃), which accounted for 93.66% *enterococcus* and 2.68% other microbial community (such as *Lactobacillus*, *Bacillus*, *Lactococcus*, and *Trichococcus*), was enriched via an ALE strategy [96]. DUT50 tolerated inhibitors (up to 9.74 g/L) derived from dilute sulfuric acid pretreatment of corn stover and also showed efficient C5 and C6 sugars utilization in the undetoxified hydrolysate without experiencing CCR effect, leading to 71.04 g/L LA production with a yield of 0.49 g/g corn stover via SSCF process. In another study, a novel synthetic microbial consortium was also constructed based on a combination of a detoxification engineered *Pseudomonas putida* KT2440 and a LA-producing *B. coagulans* NL01 [97]. Specifically, in the first step, the engineered *P. putida* rapidly degraded diverse inhibitors of undetoxified corn stover hydrolysate pretreated by dilute acid and could also not consume the major fermentable sugars in hydrolysate due to the deletion of the sugar metabolism pathway. Then, *B. coagulans* used detoxified hydrolysate to produce LA, achieving a LA titer of 35.8 g/L with a yield of 0.8 g/g total sugars.

In addition, to obtain high concentration of cellulosic biochemicals, enzymatic hydrolysis process is another central obstacle because of the multi-process integration and high cost of cellulolytic enzymes [98, 99]. Thus, integrating multi-process steps into one single unit operation, named consolidated bioprocessing (CBP), is another promising strategy for directly cellulosic LA production [100], which can improve LA economic competitiveness using lignocellulosic biomass as a feedstock.

CBP process is usually based on synergistic microbial consortia [100, 101]. In this case, lignocellulose degradation microorganism is used to overproduce fermented sugar via secreted cellulolytic degrading enzyme, which can be further converted to produce other biochemical using engineered microbial cell factories. Some biochemicals such as organic acids and ethanol have been produced via this way. Recently, this strategy is also used for cellulosic LA production. For instance, 19.8 g/L LA was obtained via a synergistic fungal–bacterial (*Trichoderma reesei/Lactobacillus pentosus*) consortium system using non-detoxified steam pretreatment of beech wood as a feedstock [100]. In another study, Jiang, et al. developed a new synergistic fungal–bacterial (*Trichoderma asperellum/Lactobacillus paracasei*) consortium system, which can directly produce 14.9 g/L LA from corncob as a feedstock without any prior pretreatment process [101]. Thus, constructing tolerant LA strains combined with these novel synergistic microbial consortia will further improve the economic benefit of cellulosic LA production in future.

## Conclusions

In this review, we summarize the inhibitors derived from lignocellulosic biomass pretreatment and their molecular toxic mechanisms, and construction of tolerant LA strains based on microbial tolerance engineering. However, economic competitiveness challenges still exist. Fortunately, with the development of efficient technologies (such as high-throughput screening, multiplexed automated ALE systems, and CRISPR/Cas9 gene editing tool), construction efficiency of strain tolerance modification can be accelerated. In addition, microbial tolerance engineering crosslinking other novel approaches including designing biomass and synergistic microbial consortia can also further improve economic competitiveness for cellulosic LA production.

## Data Availability

We declare that all data generated or analyzed during this study are included in this article.
